# Using ncRNAs as Tools in Cancer Diagnosis and Treatment—The Way towards Personalized Medicine to Improve Patients’ Health

**DOI:** 10.3390/ijms23169353

**Published:** 2022-08-19

**Authors:** Roberto Piergentili, Giuseppe Basile, Cristina Nocella, Roberto Carnevale, Enrico Marinelli, Renato Patrone, Simona Zaami

**Affiliations:** 1 Institute of Molecular Biology and Pathology, Italian National Research Council (CNR-IBPM), 00185 Rome, Italy; 2 Trauma Unit and Emergency Department, IRCCS Galeazzi Orthopedics Institute, 20161 Milan, Italy; 3 Head of Legal Medicine Unit, Clinical Institute San Siro, 20148 Milan, Italy; 4 Department of Clinical Internal, Anaesthesiological and Cardiovascular Sciences, “Sapienza” University of Rome, Viale del Policlinico, 155, 00161 Rome, Italy; 5 Department of Medico-Surgical Sciences and Biotechnologies, “Sapienza” University of Rome, 04100 Latina, Italy; 6 Mediterranea Cardiocentro-Napoli, Via Orazio, 80122 Naples, Italy; 7 PhD ICTH, University of Federico II, HPB Department INT F. Pascale IRCCS of Naples, Via Mariano Semmola, 80131 Naples, Italy; 8 Department of Anatomical, Histological, Forensic and Orthopedic Sciences, Section of Forensic Medicine, “Sapienza” University of Rome, 00161 Rome, Italy

**Keywords:** microRNA, miR, oncogene, oncosuppressor, gene therapy, epigenetics, Europe’s beating cancer plan

## Abstract

Although the first discovery of a non-coding RNA (ncRNA) dates back to 1958, only in recent years has the complexity of the transcriptome started to be elucidated. However, its components are still under investigation and their identification is one of the challenges that scientists are presently facing. In addition, their function is still far from being fully understood. The non-coding portion of the genome is indeed the largest, both quantitatively and qualitatively. A large fraction of these ncRNAs have a regulatory role either in coding mRNAs or in other ncRNAs, creating an intracellular network of crossed interactions (competing endogenous RNA networks, or ceRNET) that fine-tune the gene expression in both health and disease. The alteration of the equilibrium among such interactions can be enough to cause a transition from health to disease, but the opposite is equally true, leading to the possibility of intervening based on these mechanisms to cure human conditions. In this review, we summarize the present knowledge on these mechanisms, illustrating how they can be used for disease treatment, the current challenges and pitfalls, and the roles of environmental and lifestyle-related contributing factors, in addition to the ethical, legal, and social issues arising from their (improper) use.

## 1. Introduction—Filling the Protein World with RNA

Some decades ago, it was assumed that the biological needs of cells were essentially met through the actions of proteins. This assumption came basically from the experiments of Beadle and Tatum, who ca. 80 years ago for the first time showed the direct link between genes and enzymatic reactions in the organism *Neurospora crassa* [1]—the so called *one gene*, *one enzyme* hypothesis—whose discovery earned them the Nobel Prize in Physiology or Medicine in 1958, together with Lederberg. This concept was further expanded thanks to Vernom Ingram’s work in 1956, with the statement *one gene*, *one polypeptide*, when by studying the sickle cell hemoglobin he found that genetic variations in proteins could affect only a single polypeptide chain inside a multimeric protein complex [2]. The discovery of the DNA structure [3] and the cracking of the genetic code in the following years [4] set the basis for the formulation of the central dogma of molecular biology, first formulated in 1956 [5] in the following form: “once information has got into a protein it cannot get out again”. Beyond the obvious consequence of information not travelling back from proteins to nucleic acids, the central dogma indirectly tells us two additional things: (1) the flux of information cannot go beyond the protein level, which means that once the information arrives at a protein, the protein performs the cellular job; (2) however, nothing prevents information from stopping “before” reaching a protein, which in turn means that the information can be used by other molecules, i.e., DNA and RNA. As for the DNA, there are portions of the genome that store information that is strictly connected to the DNA; indeed, this part of the genome is normally not transcribed at all—for example, in centromeres and telomeres, in which (i) the information is stored in the DNA sequence itself; (ii) the cellular jobs are essentially chromosome segregation and integrity, respectively [6]; and (iii) their maintenance is epigenetically regulated [7]. As for the RNA, the first identification of tRNA (transfer RNA) dates back to 1958 [8], and in the same year the ribosome components started to be identified [9], including ribosomal RNA (rRNA). Together, the tRNAs and rRNAs represent more than 95% of the total mass of the RNA inside a cell [10]; rRNAs derive from approximately 300–400 gene repeats organized in 5 clusters per human haploid genome [11], producing millions of rRNA molecules per cell, while tRNAs are transcribed by ca. 500 genes in *H. sapiens* [12], producing a few million transcripts.

Interestingly, “non-coding” RNAs were discovered before the coding ones, i.e., mRNAs (messenger RNAs), the molecules physically conveying information between DNA and proteins, whose identification occurred in 1961 [13,14,15]. Starting from the 1970s in the twentieth century, several additional non-coding RNAs (ncRNAs) were identified, either as single molecules performing a specific task (such as *Xist*, *TERRA*) or entire categories performing similar tasks and sharing common structural characteristics; an incomplete list of these molecules includes the following (in parentheses is the abbreviated name if present and the approximate year(s) of first discovery): small nuclear RNA (snRNA, 1977), transfer RNA-derived small RNA (tsRNA, 1977-79), ribozymes (1980), Y RNA (1981), antisense RNA (1981-86), interfering RNA (1990), *Xist* (1992), small nucleolar RNA (snoRNA, 1992), microRNA (miRNA, 1993), *Tsix* (1999), riboswitches (2002), Piwi-associated RNA (piRNA, 2006), *TERC* (2007), *TERRA* (2010), enhancer RNA (eRNA, 2010), circular RNA (circRNA, 2012), and ribosome-associated non-coding RNA (rancRNA, 2012) (reviewed in [16,17,18,19,20,21,22,23]). It is now clear that the transcriptome largely outsizes the proteome in terms of the number of different molecules: a large part of the human genome is transcribed into RNAs, but the protein-coding loci account for just 3% of it [24]. To further complicate this scenario, in recent years even the dichotomy of coding vs. non-coding RNAs has been weakening, due to the discovery of bi-functional RNAs [25], which are RNA molecules that have an open reading frame (ORF) but at the same time can also fulfill other cellular functions without being translated.

## 2. Overview of Non-Coding RNAs: Abundance, Types and Classification

In the easiest scenario, non-coding RNAs (ncRNAs) are generally defined by the absence of an ORF in their sequence. This class of RNAs is largely the most abundant in the cell, exonic sequences covering a mere 1% of the total human genome [26]. Since the first human genome draft [27], it has been clear that for the most part such RNAs could not be just a background of the ORF transcription; in fact, the human genome contains approximately 20,000 protein coding genes, while transcripts come from the activity of ca. 93% of the human genome, with 53% of them coming from regions outside the gene boundaries (intergenic sequences), thereby exceeding the 120,000 non-coding transcriptional units [26,28]. However, an exact estimate of their number is extremely hard to obtain because a locus may encode for more than one ncRNA (up to dozens in a row in the case of microRNAs), but not all have a biological function. For example, miRs derive from a precursor double-stranded RNA, but while in some cases only one strand is biologically active, in other cases both strands are retained in the cell and perform different functions. Moreover, it is also likely that a significant portion of them are devoid of any biologically relevant function, and indeed are mere byproducts of the transcription of nearby sequences [10].

**Figure 1:**
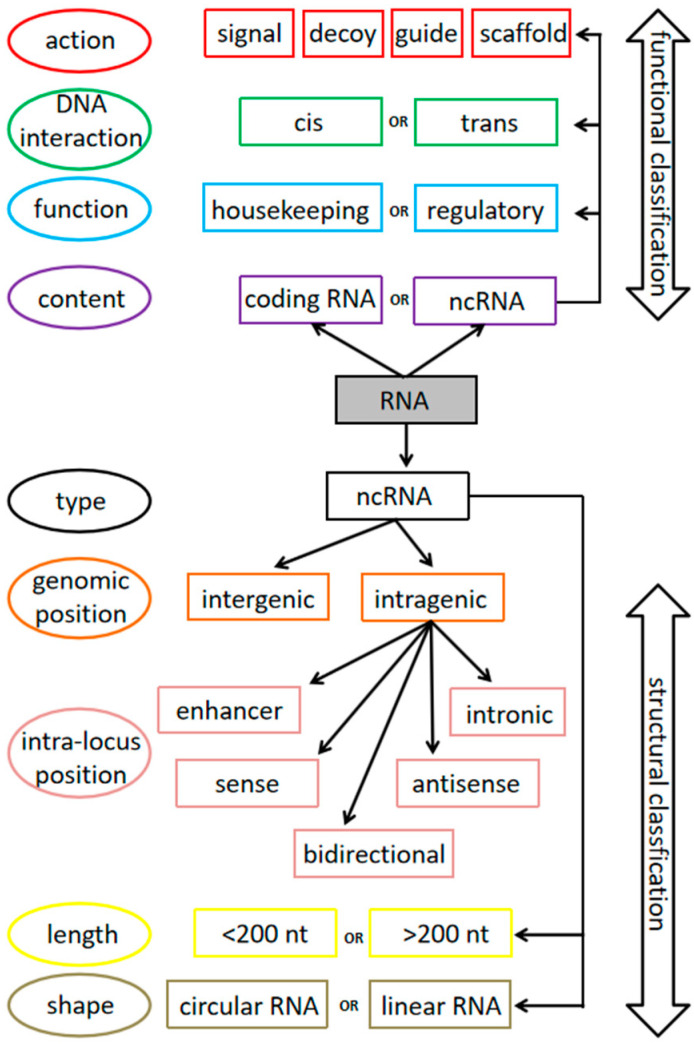
Classification of non-coding RNAs. Due to their highly heterogeneous nature, ncRNAs are classified according to several distinct variables. Although the most common parameter is their length, several other classifications are used, according to the context in which they are described. In general, the most common classifications rely either on functional aspects (**top**) or on the basis of structural features (**bottom**), as indicated by the double-headed arrows on the right. The different means of classification are depicted in the left column of the figure, inside the circles, while the corresponding RNA denominations are inside squares. Means and denominations are indicated in matching colors to ease the figure readability. To further complicate this scenario, any ncRNA can be assigned to more than one of the illustrated boxes. For example, MALAT1 (see text) is at the same time long, linear, sense, trans, and regulatory in nature [29].

The ncRNAs represent a highly heterogeneous group (Figure 1). Because of this, they are arbitrarily classified into two broad categories according to their length, with a threshold of approximately 200 nucleotides (nt). Those below the threshold are called short ncRNAs (sncRNAs), and in most cases their length is below 30 nt; those above the threshold are named long ncRNAs (lncRNAs), and may be as long as several kilobases [29]. The sncRNA group includes subgroups such as microRNAs (miRs or miRNAs), which recognize and bind partially complementary sequences located in other RNAs, either coding or non-coding, altering protein expression; Piwi-interacting RNAs (piRNAs), which function mainly in the germ line and inhibit the transcription and movement of retrotransposons, retroviruses, repetitive sequences, and other mobile elements; small interfering RNAs (or short interfering RNAs, siRNAs), double-stranded RNA molecules that promote target mRNA degradation but also play a role in antiviral activity and chromatin remodeling; small nuclear RNAs (snRNAs), involved in pre-mRNA splicing; and small nucleolar RNAs (snoRNAs), involved in RNA modification [30,31]. A comparable classification for lncRNAs is not possible, due to their ample variability in terms of their genome position (intragenic, intergenic), direction of transcription (sense, antisense), length (starting at around 200 nt and up to several kb), function (acting as transcriptional or translational regulators, chromatin modifiers, enhancers, decoys, ceRNAs, micropeptide templates, etc.), structure (linear, circular), cellular localization (nucleus, cytoplasm), and so on [28,32].

## 3. Competing Endogenous RNA Networks (ceRNETs): When lncRNAs and sncRNAs Interact

**Figure 2:**
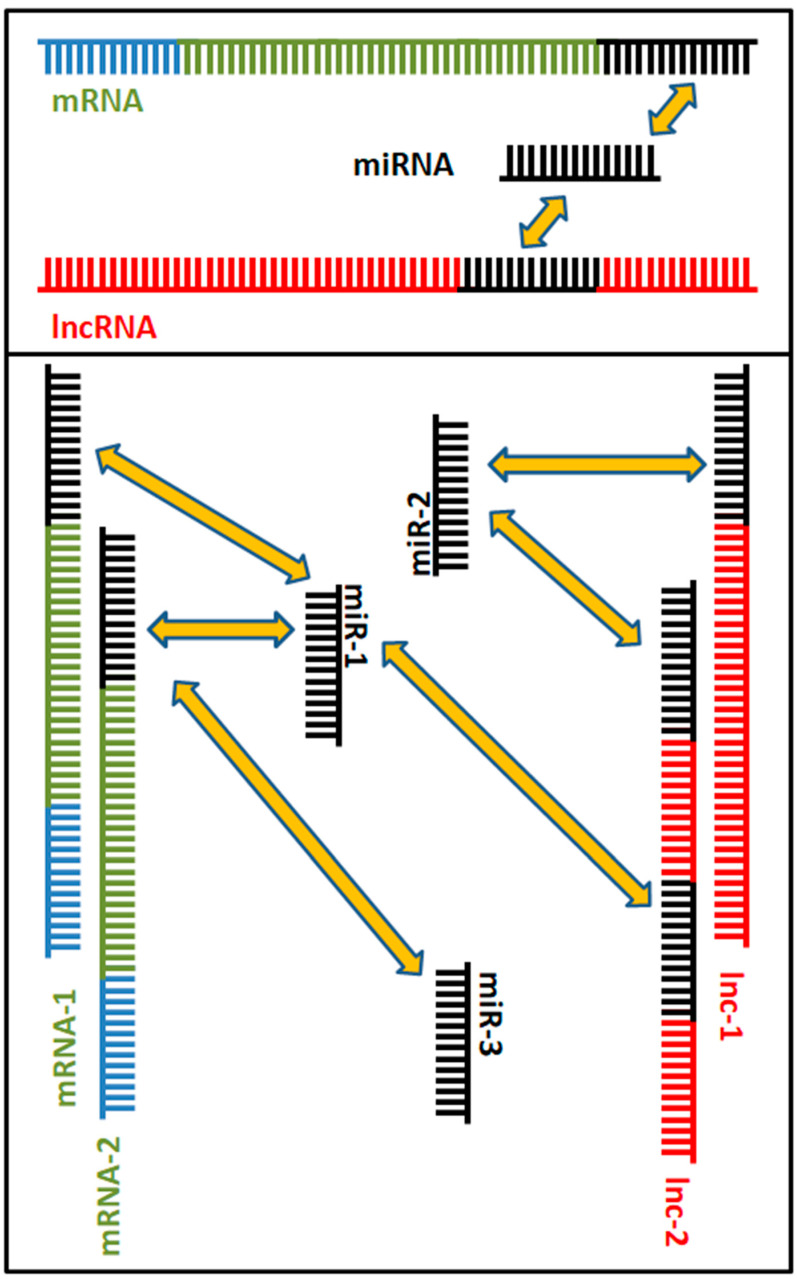
Competing endogenous RNA network. **Top panel:** In the easiest (and less common) situation, a basic ceRNET is composed of three actors: the mRNA, lncRNA, and miR (or miRNA). The interactions among them are sketched with the orange arrows. The long RNA molecules compete for the binding of the miR, and the relative concentration of these two decides the fate of target gene expression. If the concentration of the lncRNA is higher, all miR molecules are sequestered (sponged) and the mRNA can be translated into a protein; instead, if the lncRNA concentration is low, miR molecules can bind the target mRNA (usually at their 3′-UTR end), promoting either its degradation or translation block. The binding occurs thanks to sequence homology (black sequences). Additional color codes: blue is the mRNA 5′-UTR; green is the mRNA coding sequence; black is the mRNA 3′-UTR, the miR, and the region of homology on the lncRNA; red is the part of the lncRNA that does not take part in the competition. For the sake of simplicity, the length of the described sequences is not in scale. **Bottom panel:** in most cases, the competition is far more complex because of multiple interactions occurring at the same time. The same miR can target more than one mRNA (miR-1 targets both mRNA-1 and mRNA-2); an mRNA can be bound by more than one miR (both miR-1 and miR-3 bind mRNA-2); an miR can be sponged by more than one lncRNA (miR-2 can bind both lnc-1 and lnc-2) and a lncRNA may bind multiple miRs in different places (lnc-2 binds both miR-1 and miR-2). The sum of all these contemporary interactions drives gene expression. Color codes are the same as used in the top panel.

Other than the abovementioned classification, in recent years a new category of ncRNAs has been identified based on functional assays. It has been repeatedly shown that miRs are able to interact not only with their target mRNA, but also with lncRNAs (Figure 2). In other words, the mRNA and lncRNA “compete” for the binding of the miR. On this basis, competing endogenous RNAs (ceRNAs) have been named to indicate this interaction. In this scenario, the lncRNA acts as a sponge for the miR and prevents its action on the mRNA, allowing its expression at the protein level. The deregulation of such interactions may cause alterations in cell homeostasis and be a cause of disease with an epigenetic basis. This deregulation has been found in several human diseases, including cardiovascular anomalies [33], neurodegenerative disorders [34,35], and various types of cancer [36,37,38], such as those of the urogenital apparatus [39,40,41,42]. This has a deep effect in cancer; in fact, if the target mRNA encodes for an oncosuppressor, the miR that targets it acts as an oncogene (because it inhibits the expression of an oncosuppressor), and in turn the lncRNA that sponges the miR acts as an anti-oncogene (and functionally as an oncosuppressor as well); the same logic but with opposite effects applies if the mRNA is an oncogene. This represents a further step in gene expression control at the translational level in eukaryotic cells.

The binding of the miR onto the mRNA occurs at the 3′UTR of the messenger, while the interaction between miRs and lncRNAs may also occur in other regions [43]. This creates a circuit in which the increase in cellular concentration of an miR represses the translation—and hence, the expression—of its target mRNA; instead, the increase in concentration of the competing lncRNA allows this molecule to act as a sponge for the miR, decreasing the miR–mRNA interaction, and in turn promoting mRNA translation, i.e., protein expression. However, things are more complex than this. In fact, any given miR may have several target mRNAs, an mRNA may be bound by more than one miR, and a given lncRNA may sponge several different miRs. As a consequence, metabolic pathways under ceRNA control are usually very complex and ceRNAs create a complex system of crossed interactions called ceRNA networks (ceRNETs) [44,45]. Thus, a ceRNET may be represented as a network composed of several subnetworks, where nodes are ceRNAs (lncRNAs and mRNAs), while miRs represent their connections [46,47]. This complex organization allows the cell to fine tune the mRNA expression due to these intricate relations, and at the same time the deregulation of even one of the actors in this network may impair the function of several target molecules, causing disease. In physiological conditions, the optimal control and best tuning of ceRNETs occur when the miR and interacting lncRNA are at equimolar concentrations [48], so small differences in their amounts may drive cell metabolism; instead, an evident imbalance of this equilibrium is typical of disease when one of the two mRNA controllers (either the miR or lncRNA) is over-expressed or depleted. It is then reasonable to assume that such networks can be influenced in order to diagnose and treat human conditions.

## 4. Effects of Lifestyle on ncRNA Expression and Cancer

The efforts aimed at identifying the genetic and epigenetic causes of cancer are enormous, yet it should be borne in mind that the human genome can be considered the main cause of this disease only in a minority of cases. Internal factors, such as mutations in genes, hormone imbalances, or immune system-related conditions can account for only 5–10% of cancer cases; the remaining can be directly related to external factors, such as tobacco or alcohol consumption, dietary factors, infections, and how these factors interact with the genetic and epigenetic variability of humans [49]. In this perspective, understanding the genetic background of a patient, and placing this into the environmental context in which they live, is crucial for switching from traditional to personalized medicine.

### 4.1. Tobacco and Alcohol

Tobacco smoking has long been associated with several types of cancer, either due to direct contact of the tissues with the over 70 carcinogenic chemicals produced [50] (oral, head and neck, esophagus, and lung cancers) or after their penetration into the blood stream, mainly through the lungs (liver, bladder, pancreas, stomach, bowel, cervix and ovary cancers, leukemia). Despite the advent of smokeless tobacco and e-cigarettes, the situation has not significantly improved, since most carcinogens are still present in these products [51,52]. For example, it has been shown that e-cigarettes can alter the user’s epigenome [51] and their aerosol exposure could lead to the dysregulation of hundreds of miRNAs, such as miR-126 [52]. Moreover, the chemicals contained in the liquid—especially nicotine and its derivatives—have been associated with the dysregulation of several other sncRNAs and of their target mRNAs, including miR-33, miR-330, and miR-10b [53], miR-506 [54], miR-9 and miR-101 [55], miR-622 [56], miR-133b and miR-206 [57], miR-21 [58], miR-200c [59], and miR-30a and miR-379 [60]. Notably, all of them have been linked to neoplastic transformation or tumor progression in various human cancers. Several other chemicals present in e-cigarettes may potentially alter the miR expression as well [51]; however, the direct evidence of their action through this method of administration needs further investigation. Similarly, the evidence is growing regarding the role of the smoke-related dysregulation of lncRNAs, such as CCAT1 [61], linc-RoR [62], linc00152 [62], linc00460 [63], LCPAT1 [64], linc00673 [65], H19 [66], and lncAC007255.8 [67]. In addition to smoking, alcohol consumption, another well-established cause of cancer, has been reported in a relatively high number of patients [68]. Chronic alcohol abuse has been linked to cancer in various organs, either by direct interactions with the upper aerial and digestive ways (oral cavity, pharynx, hypopharynx, larynx, and esophagus) and lower digestive tract (stomach, bowel) or by indirect effects on more distant organs, such as the liver, pancreas, and breast. Also in the latter case, several ncRNAs, either long [69,70,71] or short [72,73], have been found to be altered. Specific research on individual lncRNAs has found a correlation between drinking habits and cancer; examples include linc01133 [74] and AC012456.4 [75]. Notably, some studies are specifically focused on ceRNETs. For example, Du and collaborators recently published a study, performed in silico on data available in public databases, aimed at identifying deregulated ceRNETs in esophageal cancer (EC) [76]. They found at least four possible candidate gene modules deemed to be closely related to EC progression. Although these are only predictions, they provide a compelling framework for the further analysis of these mechanisms in lifestyle-related cancer formation. Other than alcohol, diet has long been known as a major factor in cancer [76]. In fact, it is estimated that up to 35% of cancers deaths in USA are caused by dietary factors, although such an estimation varies considerably among different countries and cultures [77]. Several chemicals can reportedly cause such effects, including nitrates, nitrosamines, pesticides, and dioxins, either ingested accidentally or being part of food additives. Because of their extremely high heterogeneity in their composition, method of action, and routes of intake (air, water, food, skin contact) [78,79,80], such aspects fall beyond the scope of this review here. Looking at the relationship among food, cancer, and epigenetics, numerous interesting findings collected over the years show the importance of food intake and eating habits in preventing cancer [81,82]. The importance and beneficial potential of some plant-based foods and compounds in cancer prevention has in fact long been researched and documented.

### 4.2. Phytochemicals

#### 4.2.1. Curcumin

Curcumin is a molecule belonging to the family of phenols; it comes from the rhizomes of turmeric (*Curcuma longa*). It has widely been used as a spice for Asian recipes and as a drug in traditional Indian (Ayurveda) and Chinese (TCM) medicine for centuries. Its properties include the inhibition of cell proliferation, invasion, migration, angiogenesis, and inflammation; in addition, it also promotes cell cycle arrest and apoptosis on various cancers, such as breast, cervical, oral, gastric, melanoma, pancreatic, colon, and prostate cancers [83]. Moreover, curcumin has been shown to exert its functions through the regulation of miR expression. In breast cancer, it acts by upregulating miR-34a [83], miR-132 and miR-502c [84], miR-181b, miR-34a, miR-16, miR-15a, and miR-146b-5p, and by downregulating miR-19a and miR-19b [85], while in recent studies several other miRs were added to the list, either involving curcumin or its synthetic analogs [86,87,88]. In gastric cancer cells, similarly to breast cancer, curcumin enhances miR-34a expression [89] but inhibits miR-21 [90], which has also been reported in other cancer types (see below); in lung cancer it downregulates miR-186 [91] and circ-PRKCA [92] but upregulates miR-142-5p [93], miR-206 [94], and miR-192-5p [95]; in chronic myelogenous leukemia curcumin induces the miR-21-mediated modulation of the PTEN/AKT pathway, causing the inhibition of leukemic cell growth, both in vitro and in vivo [96], while in acute myeloid leukemia it inhibits the expression of the lncRNA HOTAIR and enhances the expression of miR-20a-5p [97]; in multiple myeloma it upregulates miR-101, thereby inhibiting EZH2 expression [98]; in colon cancer it downregulates both miR-130a [99] and miR-491 [100] but upregulates miR-137 [101], miR-200c [102], and miR-409-3p [103]; in melanoma it enhances the expression of miR-222-3p [104]; in pancreas cancer cells curcumin downregulates miR-199a and upregulates miR-22 [105]; in human prostate cancer stem cells, curcumin influences the expression of both miR-143 and miR-145 [106,107], and similarly to breast and gastric cancer it upregulates miR-34a [108]; in ovarian cancer, a curcumin derivative (ST09) deregulated the miR-199a-5p/DDR1 axis [109], while curcumin itself upregulates the lncRNA circ-PLEKHM3, promoting the intracellular depletion of miR-320a and suppressing cell proliferation and enhancing apoptosis [110]; in hepatocellular carcinoma it downregulates the expression of circ_0078710 (and consequently enhances miR-378b expression) [111] and downregulates miR-21-5p [112] and miR-21 [113]; in renal carcinoma, curcumin acts on the circ-FNDC3B/miR-138-5p/IGF2 axis [114]; in lymphoma, miR-28-5p is upregulated by curcumin treatment [115] while miR-21 is repressed [116]; in nasopharyngeal carcinoma, curcumin regulates the circRNA_102115/miR-335-3p/MAPK1 pathway [117], and other circRNAs have been identified as well [118]; in osteosarcoma it downregulates miR-21 [119]; in glioma, curcumin regulates the intracellular amounts of both the lncRNA H19 and miR-675 [120]; in bladder cancer it downregulates miR-1246 [121]. All together, these data show the enormous potential of curcumin as an anticancer agent, also thanks to the multiple ncRNA targets and the wide array of potentially treatable cancers.

#### 4.2.2. Garcinol

Another phenolic compound and Ayurveda medical component of vegetable origin is garcinol (camboginol), another Indian spice isolated from the kokum tree (*Garcinia indica*) and used in food consumption. The first relationship among garcinol, human cancer treatment, and ncRNA was identified in relatively recent times—10 years ago—meaning the data, although promising, still require further validation. In breast cancer, garcinol reverses the epithelial-to-mesenchymal transition (EMT) through its action on miR-200b, miR200c, and let-7 [122]; in pancreatic cancer, garcinol enhances the efficiency of gemcitabine treatment by modulating a number of miRs (miR-21, miR-196a, miR-495, miR-605, miR-638, and miR-453) and promoting apoptosis [123], and similarly to what happens in breast cancer, it upregulates several miRs, including miR-200c [124]; in lung cancer it inhibits the EMT through the upregulation of various miRNAs, such as miR-200b, miR-205, miR-218, and let-7c [125]; in glioblastoma, garcinol suppresses the actions of STAT3 and STAT5A thanks to the upregulation of miR-181d [126].

#### 4.2.3. Genistein

Genistein (prunetol) is a flavonoid compound and a phytoestrogen extracted from the dyer’s broom, *Genista tinctoria*; it is present in several foods of vegetable origin, including lupin, fava beans, soybeans, kudzu, psoralea, and coffee. Also in this case, in the last years several connections have been found linking cancer, ncRNA expression, and genistein assumption. In kidney cancer cells, genistein lowers miR-21 [127], miR-23b-3p [128], and miR-1260b [129] expression. A similar action on miR-1260b is exerted also in prostate cancer [130], where genistein also downregulates miR-151 [131], miR-221, miR-222 [132], and miR-223 [133] but upregulates miR-34a, miR-574-3p, and miR-1296 [134,135,136] and enhances the expression of miR-200c and miR-141 by promoting the demethylation of the CpG sites closest to the miR-200c/miR-141 loci [137]; miR-27a downregulation by genistein is a hallmark in uveal melanoma (C918) [138], pancreatic cancer [139], lung cancer [140], and ovarian cancer (SKOV3) cells [141]. In breast cancer cells, genistein suppresses miR-155 expression and acts as an antiproliferative and pro-apoptotic molecule [142] but promotes the expression of miR-23b, causing a similar effect on cells [143]; in lung cancer it regulates the circ_0031250/miR-873-5p/FOXM1 axis [144]; in head and neck cancer, it can block the EMT by activating the miR-34a/RTCB axis [145]; in retinoblastoma cells, genistein promotes apoptosis by upregulating miR-145 [146]; in multiple myeloma cells, it upregulates miR-29b, thereby halting cell proliferation [147]; in pancreas cancer cells, it upregulates miR-34a [148], miR-200, let-7 [149], and miR-146a [150]. Genistein has been also tested in isoflavone mixtures, showing that the G2535 mixture (70.54% genistein, 26.34% daidzein, 0.31% glycitein) downregulates miR-221 in pancreas cancer cells [151].

#### 4.2.4. Epigallocatechin-3-Gallate (EGCG)

EGCG is the major polyphenol compound present in green tea. In hepatoma, EGCG enhances the cancer cell sensitivity to ionizing radiation treatment via miR-34a/Sirt1/p53 signaling pathway regulation [152]; in hepatocellular carcinoma, the tumor suppressors let-7a and miR-34a are upregulated [153], and in HepG2 cells it has been shown that this molecule acts on several miRs, causing either their up- (13 miR) or down- (48 miR) regulation [154]. A similar situation occurs both in neuroblastoma cells, where oncogenic miRs are downregulated and oncosuppressor miRs are upregulated [155], as well as in nasopharyngeal carcinoma CNE2 cells, where a total of 66 signaling pathways, primarily involved in cancer development and lipid and glucose metabolism, were shown to be regulated by EGCG-specific miRNAs [156]; in oral squamous cell carcinoma cells, EGCG significantly inhibits the proliferation rate and self-renewal capacity by upregulating miR-204 [157]; in lung cancer cells, EGCG downregulates miR-98-5p and miR-125a-3p, thereby promoting apoptosis via the enhancement of the effects of cisplatin [158]; in prostate cancer cells it increases miR-330 (an oncosuppressor) and contemporarily inhibits miR21 (an oncomir) [159]; in gastric cancer it regulates the LINC00511/miR-29b/KDM2A axis [160]; in breast cancer it promotes apoptosis by downregulating miR-25 [161]; in colorectal cancer cells, EGCG enhances the sensitivity to 5-FU by inhibiting the GRP78/NF-κB/miR-155-5p/MDR1 pathway [162]; in lung cancer it inhibits cancer stem cell-like properties by targeting mir-485-5p/RXRα [163].

#### 4.2.5. Resveratrol

Resveratrol (3,5,4-trihydroxystilbene) is a phenol produced by several plants in response to injury or infection; in food, it can be found in the skins of grapes, blueberries, raspberries, mulberries, and peanuts. It mainly acts as a strong antioxidant, and in general it promotes or enhances apoptosis in several types of cancer. Several papers exist linking this molecule to ncRNA expression and cancer. In the lung cancer A549 cell line, it has been shown that resveratrol influences the regulation of tens of miR [164], and comparable numeric results were obtained in colon and prostate cancer cells [165]. Venkatadri and collaborators found the upregulation of miR-122-5p, miR-542-3p, miR-16, miR-141, miR-143, and miR-200c in breast cancer [166]; miR-21 downregulation characterizes resveratrol’s effects in pancreatic cancer cells [167], as it blocks the malignant behavior of gastric cancer cells by downregulating miR-155-5p [168] and altering the expression of several lncRNAs, including MEG3, PTTG3P, GAS5, BISPR, MALAT1 and H19 [169]. The lncRNAs are also targets in HT-29 colon adenocarcinoma cells, where it has been found that the downregulation of CCAT1, CRNDE, HOTAIR, PCAT1, PVT1, and SNHG16 occurs [170], which lowers the levels of miR-3687 and miR-301a-3p while upregulating miR-3612 in TGF-β-induced HT-29 cells [171]. In liver cancer, it is able to suppress several malignant phenotypes through miR-185-5p upregulation [172]. Beyond the other compounds described here, resveratrol is able to upregulate miR-34a to suppress the proliferation, induce the apoptosis, and inhibit the invasion and migration of OV-90 and SKOV-3 ovarian cancer cell lines [173]; in skin squamous cell carcinoma, resveratrol inhibits proliferation, migration, and invasion through upregulating miR-126 [174]; in malignant melanoma cells, resveratrol induces apoptosis by regulating the miR-492/CD147 pathway [175]; in osteosarcoma, resveratrol blocks the tumor progression via miR-139-mediated NOTCH1 regulation [176].

#### 4.2.6. Quercetin

Quercetin (3,3′,4′,5,7-pentahydroxyflavone) is a bioflavonoid found in fruits (mainly citrus), plant seeds, grains, olive oil, apples, kale, capers, and onions; variable amounts can also be found in beverages and seasonings, such as beer, wine, and vinegar. In pancreatic cancer cells, quercetin upregulates let-7c, thereby inhibiting cancer progression [177]; in gastric cancer cells, quercetin upregulates miR-143 [178]; in lung cancer cells, quercetin upregulates miR-16 [179] and promotes radio-sensitivity through the overexpression of miR-16-5p [180]; in ovarian carcinoma cells it acts by upregulating the expression of microRNA-145 [181]; in breast cancer cells it inhibits proliferation and invasion by upregulating miR-146a [182]; in hepatocellular carcinoma cells, quercetin promotes apoptosis by activating the p53/miR-34a/SIRT1 signal feedback loop [183]; in osteosarcoma cells, quercetin enhances the toxic effects of methotrexate by decreasing, among others, the anti-apoptotic miR-223 [184]; in triple negative breast cancer cells, a methoxylated quercetin glycoside isolated from *Cleome droserifolia* is able to repress the cellular proliferation, colony-forming ability, migration, and invasion capacities by modulating a ceRNA network, where it reduces the oncogenic lncRNA MALAT-1 and induces TP53 and its downstream miRNAs, miR-155 and miR-146a [185]. Interestingly, the same substance from this plant is also able to limit the cellular viability and anchorage-independent growth of hepatocellular carcinoma cells in a TP53/miR-15/miR-16-dependent manner [186]. In esophagus cancer cells, quercetin inhibits growth and metastasis by modulating the miR-1-3p/TAGLN2 pathway [187]; in lung cancer, quercetin inhibits the survival, proliferation, migration, and invasion of NSCLC cells and enhances their apoptosis by targeting the lncRNA SNHG7/miR-34a-5p pathway [188]; in oral squamous cell carcinoma it significantly suppresses the proliferation and invasion of CAL-27 cells in a dose-dependent manner, while upregulating the miR-1254/CD36 cascade [189]; in HBL-52 meningioma cells, quercetin promotes apoptosis by over-expressing miR-197 [190].

#### 4.2.7. Other Compounds

Tens of other natural substances have been studied over time for their action on ncRNA expression in cancer, but to date there are limited data available as to their method of action. Readers interested in broadening their knowledge of such correlations can draw upon the currently available specific research studies [191,192,193,194,195].

### 4.3. Obesity

Obesity is a complex, multifactorial condition that is caused by the interaction of genetic, metabolic, social, behavioral, and cultural factors. Obesity has a significant impact on health, psychosocial well-being, life expectancy, and quality of life. The multiple components of this condition do not allow one to group patients together, beyond the common BMI (body mass index, kg/m^2^) being over a set threshold, which is, however, a very limited criterion [196]. For this, subclassifications of obesity exist to reflect its complexity [197]. The spectrum of diseases linked to obesity are equally complex, and frequently associated with specific geographic regions [198]. The most common conditions associated with obesity are diabetes, hepatic steatosis, cardiovascular diseases, stroke, dyslipidemia, hypertension, gallbladder problems, osteoarthritis, sleep apnea, and other breathing problems; in addition, obese people also show an increased risk of getting some types of cancer, such as endometrial, breast, ovary, prostate, liver, gallbladder, kidney, and colon cancers [197]. It is widely accepted that this increased risk is linked to chronic inflammation caused by excessive weight [199], although several other links can be drawn, such as microbiome alterations, diabetes, and an altered steroid metabolism [200]. Losing weight and keeping it off, through a diet with nuts, fruits, vegetables, and olive oil; increasing physical exercise; and cutting down on alcohol consumption are all known to enhance life quality, reduce cancer risk, and improve health overall [201]. Several lncRNA have been shown to be involved in adipogenesis and lipid homeostasis [202,203]. In mice, a specific regulation of lncRNAs by nutrients, hormones, and transcription factors in vitro has been highlighted [204], and circulating lncRNAs in obese patients are different from those in controls [205]. Interestingly, some of these lncRNAs (such as ANRIL, H19, and HOTAIR) are also dysregulated in cancer, creating a link between the ncRNA expression profile and cancer risk in obese people, as reported by Yau and colleagues [206]. As expected, this is equally true also for sncRNAs (mainly miRs). A recent study compared the ncRNAs in obese people, colorectal cancer patients, and healthy controls, showing that there is a significant overlap in dysregulated ncRNAs in obese people and cancer patients [207]. Moreover, another group showed that dysregulated miRs (especially miR-31 and miR-215) are a hallmark of obesity, and that weight loss can change the expression profile of these patients, showing a highly dynamic response of miR expression related to weight [208]. Leptin is a hormone that is predominantly made by adipose cells and enterocytes in the small intestine; it helps to regulate energy balance by inhibiting hunger, which in turn diminishes fat storage in adipocytes. It has recently been demonstrated that exposure to leptin downregulates the expression of miR-628 and increases cell proliferation and migration in prostate cancer cells [209]. Another group showed that platelets from patients with visceral obesity can strongly promote colon cancer growth, likely via the activation of miR-19a [210], while Su and collaborators recently demonstrated that miR-27a promotes obesity-associated hepatocellular carcinoma by mediating mitochondrial dysfunction [211]. Several other reports can be found in the literature, reaching similar results. However, it is important to emphasize here that the link between oncogenic miR expression and obesity is strong, and that weight-related miR patient profiling is advisable when planning a cancer therapy.

### 4.4. Physical Activity

The research findings from various epidemiological studies have pointed to the pivotal role played by physical exercise in reducing the risk of developing cancer. Physical training has in fact been investigated as a non-pharmaceutical strategy to counter breast cancer [212,213], thanks to a wide array of benefits arising from improvements in outcomes such as muscle hypertrophy and strength levels, cardiorespiratory health, and body mass composition, all of which have been linked to an improved quality of life and reduced mortality risk in cancer patients [213,214,215]. The World Health Organization itself has highlighted the importance of structured exercise for public health, so much so that inadequate levels of physical activity have been deemed to be a major risk factor in breast and colon cancers (21–25% of cases), diabetes (27% of cases), and ischemic heart disease (30% of cases) [216,217]. Several epidemiological studies have stressed the beneficial effects of regular and moderate structured exercise (i.e., forms of exercise in adherence to international guidelines such as those proposed by the American College of Sports and Medicine), particularly in terms of protection [218,219]. Such beneficial effects involve the prevention of cancer onset (i.e., primary prevention) and prevention of relapse (tertiary prevention), as well as a degree of effectiveness against chronic degenerative diseases [220]. The mounting scientific evidence points to exercise and its ability to directly affect cancer (particularly breast tumor) through alterations in exercise-induced c-miRNA dynamics, which play a key role in the molecular interactions between skeletal muscle and cancer cells [221,222]. A 2016 study, which relied on the inbred female BALB/c mice (6–8 weeks old) model of breast cancer, showed how a 5-week exercise training protocol along with neoadjuvant hormone therapy led to higher levels of miRNA-206 and let-7a expression (both of which are linked to tumor suppression) and lower expression of the oncomiR miR-21 in cancer tissue [223]. Lower ERα and HIF-1 mRNA levels, associated with tumor growth and angiogenesis [224,225], and lower Ki67 expression (a nuclear marker pointing to cell proliferation and linked to lower survival rates in women with breast cancer) were also observed. Such findings are indeed relevant, even though the role of c-miRNAs triggered by regular exercise in breast cancer patients is still inconclusive. Such dynamics may be explained in light of the fact that the expression modulation of a rather broad array of miRNAs such as miR-1, -21, -23a, -133a, -133b, -181a, -206, -378, and -486 takes place in skeletal muscle tissue [226,227,228] and in the bloodstream [229,230] after various exercise-based approaches. The expression of miR-133a has been found to be considerably lower in five cell lines of breast cancer (MCF-7, MDA-MB-231, BT-549, SK-BR-3, and T47D) as opposed to the normal line HBL-100, and in human breast cancer tissue versus adjacent non-cancerous breast tissue. Such findings seem to point to the possibility that miR-133a can act as a systemic factor downregulating tumor progression and following physical exercise, after migrating from the skeletal muscle to the bloodstream and ultimately to cancer cells [231]. It is worth pointing out that several such miRNAs can inhibit or slow down cancer development, metastasis, and progression. Studies have highlighted noteworthy variations in the c-miR-133a-3p in high responders relative to low ones following supervised sessions of resistance training in breast cancer [232]. Moreover, alterations in the expression of c-miRNAs, lower expression levels of c-oncomiRs, and a more considerable enhancement of tumor suppressor miRNAs in the control group undergoing hormonal therapy-exercise training (aerobic exercise-based training three times per week over a 12-week period, via a high-intensity interval training protocol) were reported in a recent study [233]. Exercise-based approaches have recently been shown to impact the rno-miRNA-regulated target cancer gene candidates ITPR3, SOCS6, ITGA6, and NKX2-1 as biomarkers for cancer prognosis in rheumatoid arthritis diagnoses in pristane-induced arthritis (PIA) rat models [234]. Overall, the research points to as many as 14 miRNAs involved in pathways relevant to cancer whose expression can be modulated by regular structured exercise, while the most noteworthy effects include the different expression levels of two miRNAs that affect breast cancer progression, in addition to the already mentioned upregulation of miR-206 and downregulation of anti-miR-30c. Such effects are indeed relevant in light of the fact that miR-206 transfection and anti-miR-30c silencing can inhibit cell growth and enhance MCF-7 cells apoptosis [235,236,237]. In addition, apoptosis and induced growth arrest in the G1/S phase of the cell cycle can be further driven by the combined use of these two miRNAs, which can be assessed and used as non-invasive biomarkers for breast cancer [220,238]. The regulation of the cellular immune system constitutes another noteworthy association between cancer and exercise, as cytotoxic immune cells have been observed to be mobilized to the circulation during exercise via blood-flow-induced shear stress and adrenergic signaling [239]. Studies on animal models observed how the tumors from running mice exhibited higher mRNA expression levels of receptor ligands capable of mobilizing NK cells (namely H60a, MULT1, Clr-b), in addition to IL-2, IL-15, and IFNγ cytokines and CCL3, CXCL10, CX3CL1, and chemerin chemokines, all associated with natural killer (NK) cell activation and chemotaxis. No changes in the expression of markers of angiogenesis (i.e., CD31 and VEGF-A) were observed [240]. The cytotoxic immune cells, thus, “scan” the system in order to recognize and eliminate altered cells. A noteworthy capability for the suppression of tumor growth mediated by exercise has been reported in animal-based studies, possibly linked to the epinephrine-dependent mobilization of NK cells, followed by higher levels of immune cell infiltration into cancerous tissues [241]. The adrenergic signaling was shown to be at the heart of the exercise-induced cancerous growth suppression. Immune cell stimulation and mobilization fostered by exercise were investigated in depth in a recent study involving cancer patients, which concluded that breast cancer survivors were capable of mobilizing NK cells to the circulation to the same extent as healthy controls of the same age [240].

## 5. Analyzing ceRNETs for Diagnosis and Targeting Them for Therapy: The State of the Art

The deregulation of several ncRNAs in most—if not all—cancers is a well-known and proven fact; for example, in the abovementioned case of endometrial cancer (EC), it has been reported that hundreds of ncRNAs are potentially deregulated [242,243,244], and this holds true for all tumors investigated so far. The levels of ncRNAs in cancer are dramatically altered by stress from the tumor microenvironment. The stress conditions include defined characteristics of cancer, such as hypoxia, chronic inflammation, and the deprivation of nutrients, including some that are essential in cancer metabolism, such as glucose or glutamine [4]. The microenvironment of the tumor presents significant differences compared to healthy tissues, including in terms of oxygenation and the metabolic status. Indeed, hypoxia is a hallmark characteristic of the tumor microenvironment and plays a crucial role in growth and metastasis. Upon hypoxia, hypoxia-inducible factors (HIFs) modulate many ncRNAs [245,246], including MALAT1 [247], the lncRNA HOTAIR in non-small cell lung cancer (NSCLC) [248], and the lncRNA H19 in glioblastoma [249]. An interesting aspect of this relationship between the tumor microenvironment and ncRNAs is that it is a reciprocal relationship. If on the one hand, as described, the tumor microenvironment modulates the expression of ncRNAs, it is also true that circulating ncRNAs have the ability to strongly modulate the behavior of cells populating the tumor microenvironment, thereby remodeling the metastatic niche and eventually favoring carcinogenesis [250]. Indeed, carcinogenesis appears as a multistage process to which both exogenous and endogenous factors contribute [251,252]. The lncRNAs, and particularly circRNAs, are found to act as ceRNAs that play critical roles in the development and progression of cancers. Abnormally expressed ncRNAs may have repercussions on many processes related to tumorigeneses, such as cell proliferation, metastasis formation, and drug resistance, by regulating different intracellular pathways. In several types of cancer, most lncRNAs are either up- or downregulated. These lncRNAs favor all stages of tumor development through the promotion of mRNA expression and constancy [253], by favoring mRNA stability [254], or by modulating miR [254,255,256,257,258,259,260,261,262,263,264,265,266,267,268,269,270,271,272,273,274,275,276,277,278,279,280,281,282,283,284,285,286,287,288,289,290,291,292,293,294,295,296,297,298,299,300]. The aberrant phenotype is the result of the modulation of typical pathways playing key roles in cell survival [255,259,263,265,283], apoptosis [256,258,272,276,286,289,297], or glucose metabolism [253,294,295,300]. For example, a recent study [255] demonstrated the molecular mechanisms of action of the lncRNA named MALAT1, which was found t be upregulated in osteosarcoma. This study showed that MALAT1, via the downregulation of miR-376a, accelerates osteosarcoma via the Wnt/β-catenin pathway [255], which is a conserved signaling axis participating in diverse physiological processes such as proliferation, differentiation, apoptosis, migration, and invasion [301]. The Wnt/β-catenin signaling pathways is also activated in colorectal cancer by the lncRNA NEAT1 (nuclear-enriched abundant transcript 1), which modulates the miR-34a/SIRT1 axis [281]. Another important pathway in cancer is the phosphoinositide 3-kinase-AKT-mammalian target of the rapamycin (PI3K-AKT-mTOR) pathway, which is frequently hyperactivated in cancer and is essential for tumor cell growth and survival [61]. Indeed, several lncRNAs such as HOTAIR, HOXD-AS1, LINC00511, H19, and LINC01554, by targeting specific miRs, increase the expression of AKT and mTOR, promoting aberrant phenotypes [262,264,271,274,278,287,296].

VEGF has been proposed to serve as a crucial gene promoting angiogenesis during tumor metastasis. The lncRNA NUTM2A-AS1 (an antisense transcript) positively regulates ROS production, and finally VEGF expression, favoring gastric cancer progression and drug resistance [269]. Additionally, LINC00173.v1 in NSCLC, by downregulating miR-511-5p [270], and NEAT1 in colorectal cancer, by downregulating miR-205-5p [282], increased VEGFA expression. Circular RNAs (circRNAs) are a novel class of endogenous covalently closed RNA molecules that function as microRNA sponges. Several circRNAs were upregulated in cancer-promoting proliferation, migration, and invasion [284,285,291,293]. The deregulation lncRNAs provides important advantages in cancer diagnosis. First, this is a way to understand the mechanism of the formation of a good fraction of neoplasms for which an evident mutation in the coding sequence of a tumor suppressor gene or oncogene cannot be found. Secondly, usually only a subset of these ncRNAs is deregulated in a given tumor, and this provides a way to identify not only different tumor subtypes, but even different cell populations inside the same lesion. Third, on the basis of the altered panel of ncRNAs, and knowing or guessing (through an in silico approach) the possible mRNA targets, it is possible to identify the molecular pathway(s) altered in the transformed cells, allowing one to foresee whether a tumor can be treated with a certain drug instead of another, or to evaluate the tumor resilience to radio- or chemotherapy or the ability of the tumor to escape apoptosis or the immune system. Fourth, the analysis of the altered target genes, coupled with other investigations such as cytology and histology, may allow the oncologist to evaluate the malignancy of the tumor, as well as its chance of relapsing. With a systematic approach involving molecular biology, biochemistry, high-throughput sequencing, and artificial-intelligence-assisted data analysis, and coupling these approaches with well-established diagnostic tools currently used in the everyday medicine, the road towards personalized medicine is at hand. The identification of ncRNAs as fundamental players in gene expression raises the possibility of using them as both diagnostic markers and possible therapeutic targets [302,303,304,305,306]. Indeed, numerous clinical trials of ncRNAs are ongoing [307]. When we consider ncRNA-based therapies, we should take into account two important aspects: (1) the RNA target and (2) the delivery methods of RNA therapeutics. Regarding the first aspect, among the ncRNAs, miRNAs are the most extensively investigated as therapeutic targets. The two major therapeutic forms used are miRNA mimics and inhibitors of miRNAs, known as anti-miRs/antagomiRs. The first group are used to mimic the function of endogenous tumor suppressor miRNAs, and the latter to deplete oncogenic miRNAs. Among the miRNA mimic therapeutics, we recall here MRX34, which is a synthetic double-stranded mimic of the miR-34a and was the first miRNA-based therapy to be introduced into the clinic. In 2020, the final phase 1 results for the pharmacodynamics and determination and evaluation of the recommended phase 2 dose (RP2D) of MRX34 were reported [308]. Patients with advanced solid tumors refractory to standard treatments were enrolled to receive MRX34, with oral dexamethasone premedication, intravenously daily for 5 days in 3-week cycles. MRX34 demonstrated a manageable toxicity profile; the pharmacodynamic results showed the delivery of miR-34a to tumors and the dose-dependent modulation of target gene expression in white blood cells. The trial was closed early due to serious immune-mediated adverse events [308], indicating that although very promising, the use of these molecules in cancer therapy is still an issue, and in many cases needs deeper analyses and testing. The miRNA inhibitors include several groups, such as (1) antisense oligonucleotides (ASOs), which are single-stranded RNAs with lengths ranging from 18 to 30 base pairs (bp). They function by modifying the expression of a target mRNA, by either altering the splicing or by recruiting RNase H, leading to target degradation [309]; (2) the CRISPR/Cas system, the use of which is an innovative strategy showing robustness, specificity, and stability in the modulation of miRNA expression [310]. CRISPR genome-editing technology has been successfully used to modulate the expression of miRNAs in several types of tumors [311,312,313]. For example, Zhou et al. [311], in a hepatocellular carcinoma (HCC) cell line, knocked out miR-3188, which is markedly overexpressed in HCC tissues. They demonstrated that the miR-3188 knockdown successfully decreased cell growth, invasion, and migration [311]. The CRISPR/Cas system was also widely used to modulate the expression of lncRNAs [300,314,315,316]. Ali et al. [314] performed the CRISPR/Cas9-mediated knockout of lncRNA-RP11-156p1.3 in an HCC cell line, resulting in decreases in the cell count and viability [314]. CRISPR/Cas9 gene editing was also used to knockout lncRNA XLOC_005950, which works as a molecular sponge of hsa-miR-542-2p in osteosarcoma [300]. The results showed that the lncRNA XLOC_005950 knockout, by decreasing the PFK muscle (PFKM) activity, reduced the intracellular glucose, lactic acid content, and cell proliferation in osteosarcoma cells [300]. Other significant approaches to target lncRNAs are double-stranded RNA-mediated interference (RNAi) approaches and ASOs. For example, the effect of the knockdown of MALAT1 using ASOs was observed in a mouse model of breast cancer, the MMTV-PyMT model (mouse mammary tumor virus–polyoma middle tumor antigen), which develops spontaneous mammary tumors that closely resemble the progression and morphology of human breast cancers [317]. The MALAT1 loss results in slower tumor growth by inducing alterations in the gene expression and changes in the splicing patterns of the genes involved in differentiation and protumorigenic signaling pathways [318]. The positive effects were confirmed later by Gong and colleagues, who constructed a MALAT1-specific ASO that reduced the MALAT1 expression levels, decreased the migration ability in lung cancer cells, and significantly reduced the metastatic tumor nodule formation in vivo [319]. MALAT1 was also the target in preclinical studies with short interfering RNAs (siRNAs) to overcome the anti-androgen enzalutamide (Enz) resistance (EnzR) in castration-resistant prostate cancer. The administration of the MALAT1 short interfering RNA (10 mg/kg) for 2 weeks in xenograft mice, injected with EnzR cells, significantly suppressed the EnzR tumors [320]. Even if these RNA-based therapeutic modalities have great potential to generate a new therapeutic approach in disease in general, and in cancer in particular, to reach their full potential they first need to overcome the lipid bilayer of the cell wall to deliver RNA into cells. Indeed, the delivery methods remain the major problem to solve for the widespread development of RNA therapeutics [321]. Besides the cellular barrier, specific pharmacological barriers should also be improved. Indeed, synthetic ncRNA mimics and inhibitors generally degrade rapidly in biological fluids, absorb poorly into the intracellular space, and often may fail to reach specific target locations [305]. The delivery of drugs with nanoparticles can overcome many of these limitations. Indeed, nanocarriers encapsulate drugs and control their pharmacokinetic properties by regulating the drug release and increasing the half-life. To date, the delivery approaches with nanoparticles include lipid-based nanoparticles (LNP), polymer-based nanoparticles (PNP), and lipid–polymer hybrids. LNP are vesicles with a diameter range of 10–500 nm composed of multiple lipid layers stabilized in aqueous media by a single layer of surfactants (phospholipids, poly(ethylene glycol)-based surfactants). LNPs represent a well-established delivery system for gene therapies and are approved by the FDA for liver siRNA delivery [322]. LNPs offer several advantages, including enhanced drug stability, reduced toxicity, and control of the release rate [323,324]. Despite these promising aspects, several drawbacks remain to be addressed. For example, small molecules are encapsulated with low efficiency; moreover, cytotoxicity and systemic toxicity problems remain to be solved [324]. The other RNA delivery systems include polymer-based nanoparticles. These are between 20 and 1500 nm in particle size and made up of natural or synthetic polymers [325]. Even if they present increased stability compared to LNPs and technical advantages due to several fabrication methods, the aspects related to their toxicity have not yet been fully clarified. Finally, lipid–polymer hybrids were synthesized by adding lipids to polymeric nanoparticles, improving their delivery [326,327]. Such hybrid systems rely on the specific characteristics of lipid-based and polymer-based nanoparticles but also overcome their limits, such as their structural disintegration, limited circulation time, and loss of content [328]. Structurally, they are composed of a polymer core encapsulating the drug, surrounded by a lipid monolayer and an outer lipid–PEG layer. This structure ensures many advantages, including enhanced stability and controlled drug delivery [328,329]; however, as the use of lipid–polymer hybrids represents an innovative method, the research remains open to verifying their applicability in clinical practice. Furthermore, it is also necessary to identify lipid–polymer hybrids with the highest quality for specific uses [329]. There are several major challenges that stand in the way of treating human conditions by ncRNAs, which explains why only a very limited number of molecules are available as therapeutic agents to date. First, the choice of the target molecule is fundamental; as already mentioned, a tumor is a disease that is heterogeneous not only in different patients, but also in its cell subpopulations. Targeting one mRNA may not be sufficient to obtain relevant results. Second, the administration route is challenging as well. In some cases the therapeutic may be administered locally and directly (for example, inside a bladder cavity), but in other cases it should reach its destination through indirect routes, such as the blood flow. Third, the choice of the vector responsible for delivering the therapeutic to its target cells is far from trivial. The ncRNA may be conjugated with other molecules such as antibodies, cell-penetrating peptides (or other polymers), or metal nanoparticles; alternatively, it can be embedded in lipid nanoparticles, exosomes, or viral or mini-bacterial vectors. Each possibility has pros and cons, and deciding which one is the better in a particular situation is very complex. Fourth, the escape of the vector from the host immune system, which may recognize both the vector and the therapeutic RNA as exogenous substances and promote their degradation before they reach their target organ, may impair the whole approach. Fifth, the specificity of the target is pivotal; the vector should discriminate between healthy and sick cells inside the same organ, and frequently the adhesion molecules used by the vector to recognize their target are shared between tumor and normal cells. In addition, off-target binding to different cell types, either inside or outside the target organ, must be avoided, further complicating this setup. Sixth, the efficiency of the penetration of the vector inside the cell, a problem closely related to the preceding point, might make the therapy inefficient. Seventh, the efficacy of the therapeutic once it is inside the target celli s important; in this case, several variables should be considered—its half-life before full and possibly constant expression; the specificity of the mRNA target (avoiding off-target binding to mRNAs not involved in the disease, which is especially true for miRs and siRNAs); the use of a suitable promoter to allow sustained expression over time; and its shape (circular vs. linear, which impacts on its stability and function). Table 1 summarizes ncRNAs in terms of their tumorigenesis and drug resistance, in addition to their regulation of different intracellular pathways. In several types of cancer, most lncRNAs are either up- or downregulated. Table 2 outlines the delivery approaches via nanoparticles, including lipid-based nanoparticles (LNP), polymer-based nanoparticles (PNP), and lipid–polymer hybrids.

## 6. Ethical, Legal, and Social Issues of Personalized Medicine

Despite the potential and benefits of personalized medicine in terms of providing therapeutic options better suited to each patient’s genetic profile, a set of standards is needed to ensure the protection and fair treatment of individuals [332]. The issues concerning personalized medicine range from individual privacy to the stratification and discrimination of sub-populations based on ethnicity, equality of access, and the fair allocation of resources [333]. As such practices become mainstream, such ethical challenges need to be dealt with in order to ensure that the opportunities and benefits provided by such new scientific avenues are ethically implemented [334]. The European Union has acknowledged the importance of personalized medicine by issuing two policy papers arguing in favor of a broader use of personalized medicine (focusing on cancer diagnostics or therapeutics in particular) [335,336], while remarking that such a goal may be hampered by the still high degree of uncertainty surrounding the outcomes [337]. The key factor that can enable and unleash the full potential of personalized medicine is, according to the analysis laid out in the EU papers, an effective synergy between health data and new technologies, which is necessary to pave the way for the beneficial development of personalized medicine [338]. We can rely on its unique potential to confront cancer by means of prevention and treatment strategies enabling patients to receive the therapies that can ultimately work best for them. Such dynamics may entail considerable benefits for healthcare spending as well, since less money would be wasted on trials and ineffective treatments. For 2022, the EU plans to take further steps to harness the potential of new developing technologies such as AI, big data, and genomics through a European Cancer Imaging Initiative aimed at fostering the application of new computer-aided tools in order to improve the field of personalized medicine and provide innovative solutions [339,340]. In addition, the new Partnership on Personalized Medicine is scheduled to be launched in 2023 through funding provided by Horizon Europe, the EU’s key funding programme for research and innovation, which can tap into a budget of €95.5 billion. The partnership will aim to define priorities for research and education in personalized medicine; support research projects on cancer prevention, diagnosis, and treatment; and outline a set of recommendations for the establishment of personalized medicine approaches in clinical practice and medical research. Those goals have also been pursued by the International Consortium for Personalized Medicine (ICPerMed), launched in November 2016 [341,342]. The ICPerMed has outlined a vision for what personalized medicine will come to represent: the ultimate expression of medical evolution in the era of biotechnology and big data. Such a change, however, does call for broad-ranging adjustments and growth in the fundamental ways in which healthcare is delivered, prioritizing training and new skills for healthcare professionals and innovative tools for large-scale implementation [343]. The ICPerMed vision has been shaped and endorsed by consulting European and international experts and specialists in key areas of research, who have provided feedback on the opportunities and challenges related to personalized medicine and highlighted specific concerns and possible solutions [344]. A road map to tailored preventive strategies and approaches will be laid out by the European Commission as a preliminary step towards launching the partnership [345]. The prospect that data will likely fundamentally change healthcare has been acknowledged by established European policies, both at the individual patient level and as it pertains to the healthcare system (noteworthy in that regard is the report from the European Alliance for Personalized Medicine, “Cooperating on Data: The Missing Element in Bringing Real Innovation to Europe’s Healthcare System” [346]). It is in fact worth bearing in mind that medical records, patient information, clinical studies, and diagnostic results are but some of the data sources available in healthcare. The digitization of patient records will be an important contributor to this evolution. Big data gathered and elaborated from electronic archives will also be needed, including data from digital applications, wearable devices, and social media, providing informations on environment- and lifestyle-related factors, socio-demographics, genomics, metabolomics, proteomics, radiomics, standardized electronic health records, or precision medicine platforms [347]. An ethically and legally tenable path towards the mainstream use of personalized medicine can only be achieved by prioritizing the management of biobanking and informed consent, confidentiality [348], access to treatment, clinical translation, and direct-to-consumer genetic testing, and by putting in place measures to prevent the stratification and genetic discrimination of sub-populations based on ethnicity [349,350]. An inadequate level of genetic literacy and an inadequate understanding of personal and familial implications of germline and somatic genomic testing among patients have been cited by specialists as sources of concern arising from personalized medicine use, particularly when seeking informed consent [351]. Inequalities in terms of access are also likely to arise according to the patient’s socioeconomic status, insurance provider (or level of coverage by the national healthcare system), and cancer care facilities [352]. Although patients living in countries with publicly funded universal healthcare are less likely to be affected by access inequalities, such systems often provide coverage for procedures and treatments whose efficacy has already been established [353]. For the clinical applications of personalized medicine to be validated in terms of their efficacy, they may require larger study samples vis-a-vis conventional treatments of already acknowledged clinical value. Hence, such applications may take longer to be recognized as evidence-based [354]. The fair and equitable distribution of healthcare resources can be negatively affected by such aspects. The already cited 2022 European Union Communication [335] mentions legal and ethical standards as some of the major barriers that need to be overcome if personalized medicine and the European Digital Strategy are to be harnessed to their full capacity. Litigation cases stemming from alleged negligence and malpractice allegations [355] are in fact likely to grow as a result of personalized medicine becoming more widespread [356]. As the degree of complexity of the medical interventions grows, so does the risk that an error may do damage to the patient, leading to liability and litigation [357]. The parties that could be held accountable include the manufacturers of genome sequencers and medical devices, laboratories, pharmaceutical companies, and healthcare facilities, but most of all the doctors responsible for diagnoses and therapeutic interventions. The notion of “genetic malpractice” has been defined as the failure on the part of doctors to recommend or properly interpret genetic testing, and such dynamics can be further compounded by the still unsolved disagreements within the medical community as to the scope and timing of the implementation of genetic testing in the clinical context, or even whether such testing ought to be performed at all [343,356,357]. It is quite hard at this stage to make predictions as to how the several novel liability risks (arising from personalized medicine based on clinical genomics), which have been already explored in scientific literature [355,356], will materialize in trial courts. The outcome of such lawsuits will likely rest on the specific circumstances and facts surrounding each case, as well as the approaches put in place by plaintiffs, attorneys, experts, and judges. It is, therefore, safe to assume that the early court rulings will substantially affect the future feasibility, attractiveness, and frequency of such litigation, as both medical and legal operators will look at those rulings for guidance. Nonetheless, the need for harmonized and broadly shared legislative, regulatory, and policy standards, i.e., up-to-date clinical guidelines specifying when and where genetic testing can be useful and where it is not (at least for now), is even more transparent, in order to help guide clinical judgment, provide a degree of objectivity for judicial rulings to look at, and to partially shield doctors from malpractice lawsuits.

## 7. Conclusions

Overall, ncRNAs provide a powerful weapon against human diseases, but we are still learning how to use them. The repertoire of ncRNAs is still growing, and the process of understanding their mechanisms of action is ongoing. However, the promise of finding cures for many diseases is in sight, and the advent of new computational tools coupled with advanced massive sequencing and innovative techniques such as CRISPR-Cas9 should speed up our race towards a healthier world. At the same time, it is of the utmost importance to prioritize ethically, legally, and socially sound approaches when undertaking such innovative pathways. Personalized medicine is information-intensive in nature. The predictive, diagnostic, and therapeutic capabilities of personalized medicine rely on high-dimensionality data created using genomics and other technologies. The legal and regulatory frameworks governing such dynamics need to be adequately updated and improved so as to meet the growing challenges and unique complexities arising from the future mainstream application of personalized medicine and the vast array of technologies on which it relies. A new ethical and legal set of standards aimed at avoiding inequalities in healthcare access and genetic discrimination (which personalized medicine, with its ability to draw ever-more subtle and precise distinctions among patients, could exacerbate) is all the more necessary.

## Figures and Tables

**Table 1:** The ncRNAs involved in tumorigenesis and drug resistance.

ncRNAs	Expression	miRNAs Target	mRNAs Target	Downstream Effectors or Pathways	Aberrant Phenotype	Cancer Type	Ref.
MALAT1	Upregulated	miR-376a	NR	↑ Wnt3a/β-catenin ↓ Autophagy ↓ Oxidative stress	Proliferation Invasion Migration	Osteosarcoma	[255]
	Upregulated	miR-485-5p	MAT2A	NR	Proliferation	HPV16	[266]
	Upregulated	miR-145	SMAD3/TGFBR2	↑ TGF-β1	EMT	Prostate cancer	[277]
HOTAIR	Upregulated	/	HK2	↑ glycolysis	Proliferation Medication resistance	Lung cancer	[253]
	Upregulated	/	CCL22	↓ Immunity	Proliferation Migration Invasion	NSCLC	[330]
	Upregulated	miR-130a-3p	Suv39H1	↑ Akt/mTOR	Proliferation Metastasis	Breast cancer	[288]
	Upregulated	miR-20b-5p	RRM2	↑ PI3K–Akt	Proliferation	RB	[296]
	Upregulated	miR1/miR-206	YY1	↓ Apoptosis	Proliferation Migration Invasion EMT	Medulloblastoma	[297]
	Upregulated	miR-130a-5p	ZEB1	NR	EMT	ESCC	[298]
LINC00518	Upregulated	/	MITF	EIF4A3	Proliferation Migration Invasion EMT	Melanoma	[254]
	Upregulated	miR-335-3p	CTHRC1	↑ Integrinβ3/ FAK	Proliferation Metastasis	LUAD	[299]
XLOC_005950	Upregulated	hsa-miR-542-3p	PFKM	↑ glucose metabolism	Proliferation	Osteosarcoma	[300]
HEIH	Upregulated	miR-3619-5p	HDGF	↓ Apoptosis	Proliferation Cisplatin resistance	TSCC	[256]
	Upregulated	miR-98-5p	HECTD4	NR	Proliferation Invasion Migration	Cholangiocarcinoma	[257]
	Upregulated	miR-939	NFκB/ Bcl-xL	↓ Apoptosis	Proliferation	Colorectal cancer	[258]
HOXD-AS1	Upregulated	miR-664b-3p	PLAC8	NR	Proliferation Invasion Migration	Pancreatic cancer	[259]
	Upregulated	miR-361-5p	FOXM1	NR	Metastasis	CRPC	[260]
	Upregulated	miR-877-3p	FGF2	NR	Invasion Migration	Cervical cancer	[261]
	Upregulated	miR-186-5p	PIK3R3	↑ PI3K–Akt	EMT	Epithelial ovarian cancer	[262]
MEG3	Downregluated	miR-499-5p	CYLD	↑ E-cadherin ↓ N-caderin ↓ Cyclin D1	Proliferation Invasion	Melanoma	[263]
LINC01554	Downregluated	miR-1267	ING3	↑ Akt/mTOR	Proliferation Migration Invasion EMT	NSCLC	[264]
FOXD2-AS1	Upregulated	miR-31	PAX9	NR	Proliferation Migration	RB	[265]
	Upregulated	miR-324-3p	PDRG1	NR	Proliferation Migration Invasion	Hemangioma	[267]
	Upregulated	miR-7-5p	TERT	NR	Anoikis resistance	Tyroid cancer	[268]
NUTM2A-AS1	Upregulated	miR-613	VEGFA	↑ Oxidative stress	Cell viability Proliferation	Gastric cancer	[269]
LINC00173.v1	Upregulated	miR-511-5p	VEGFA	NR	Proliferation Migration	NSCLC	[270]
LINC00511	Upregulated	miR-126-5p miR-218-5p	COL1A1	↑ Akt/mTOR	Proliferation Migration Invasion	Lung adenocarcinoma	[271]
	Upregulated	miR-625	LRRC8E	↓ Apoptosis	Cisplatin resistance	NSCLC	[272]
	Upregulated	miR-29c-3p	NFIA	NR		Colorectal cancer	[273]
H19	Upregulated	6 miRNAs	38 mRNAs	↑ PI3K–Akt	Metastasis	Colorectal cancer	[274]
	Upregulated	miR-491-5p	ERN1	↑ LC3 ↑ Beclin	Tumor development	Glioblastoma	[331]
	Upregulated	miR-326	BCL-2	↓ Apoptosis	Leukemogenesis	Acute lymphoblastic leukemia	[276]
NEAT1	Upregulated	miR-342-3p	CUL4B	↑ PI3K-Akt	Proliferation	CSCC	[278]
	Upregulated	miR-10a-5p	SERPINE1	↑ Immune cells infiltration	Proliferation Migration	Kidney Cancer	[279]
	Upregulated	miR-23a-3p	GLS	↑ Glutamine Metabolism	Cisplatin resistance	Medulloblastoma	[280]
	Upregulated	miR-34a	SIRT1	↑ Wnt/β-catenin	Proliferation Metastasis	Colorectal cancer	[281]
	Upregulated	miR-205-5p	VEGFA	NR	Proliferation Migration Invasion	Colorectal cancer	[282]
HAS2-AS1	Upregulated	miR-137	LSD1	NR	Proliferation	Gliobastoma	[283]
circRNA hsa_circ_000 1429	Upregulated	miR-205	KDM4A	NR	Proliferation Migration Invasion	Breast cancer	[284]
circRNA hsa_circ_0000285	Upregulated	miR-582-3p	CCNB2	NR	Proliferation Migration	Hepatocellular carcinoma	[285]
	Upregulated	miR-1278	FN1	↓ Apoptosis	Proliferation	Gastric cancer	[286]
	Upregulated	miR-127-5p	CDH2	NR	Proliferation Migration	Thyroid cancer	[287]
	Upregulated	miR197-3p	ELK1	↓ Apoptosis ↓ Autophagy	Tumor growth	Cervical cancer	[289]
	Upregulated	miR-197-3p	CKS1B	NR	Proliferation Invasion	Glioma	[290]
circRNA ARAP2	Upregulated	miR-761	FOXM1	NR	EMT	Esophageal squamous cell carcinoma	[291]
circRNA-MAT2B	Upregulated	miR-431	ZEB1	↑ E-cadherin ↓ N-caderin ↓ Vimentin	EMT	NSCLC	[292]
	Upregulated	miR-610	E2F1		Proliferation	Colorectal Cancer	[293]
	Upregulated	miR-515-5p	HIF-1α	↑ glycolysis	Tumor growth	Gastric cancer	[294]
	Upregulated	miR-338-3p	PKM2	↑ glycolysis	Tumor progressione	Hepatocellular carcinoma	[295]

**Legend:** CCL22: C-C motif chemokine ligand 22; CCNB2: cyclin B2; CDH2: cadherin 2; CKS1B: CDC28 protein kinase regulatory subunit 1B; COL1A1: collagen type I alpha 1 chain; CSCC: cutaneous squamous cell carcinoma; CTHRC1: collagen triple helix repeat-containing 1; CYLD: cylindromatosis; CUL4B: cullin 4B; EIF4A3: eukaryotic translation initiation factor 4A3; CRPC: castration-resistant prostate cancer; EMT: epithelial–mesenchymal transition; ERN1: endoplasmic reticulum-to-nucleus signaling 1; ESCC: esophageal squamous cell carcinoma; FGF2: fibroblast growth factor 2; FN1: fibronectin 1; FOXM1: forkhead box M1; GLS: glutaminase; HDGF: heparin-binding growth factor; HK2: hexokinase 2; HECTD4: HECT domain E3 ubiquitin protein ligase 4; HPV16: human papillomavirus 16; ING3: inhibitor of growth family member 3; KDM4A: lysine demethylase 4A; LRRC8E: leucine-rich repeat-containing 8 VRAC subunit E; LSD1: lysine-specific demethylase 1; LUAD: lung adenocarcinoma; MALAT 1: metastasis-associated lung adenocarcinoma transcript 1; MAT2A: methionine adenosyltransferase 2A; MITF: microphthalmia-associated transcription factor; NFIA: nuclear factor IA; NSCLC: non-small-cell lung cancer; NR: not reported; PAX9: paired Box 9; PDRG1: P53 end DNA-damage-regulated 1; PFKM: phosphofructokinase, muscle; PIK3R3: phosphoinositide-3-kinase-regulatory subunit 3; PLAC8: placenta-associated 8; RB: retinoblastoma; ROS: reactive oxygen species; RRM2; ribonucleotide reductase regulatory subunit M2; SERPINE1: serpin family E member 1; SIRT1: Sirtuin 1; TERT: telomerase reverse transcriptase; TGFβ 1: transforming growth factor β 1; TGFBR2: transforming growth factor beta receptor 2; TSCC: tongue squamous cell carcinoma; VEGFA: vascular endothelial growth factor A; ZEB1: zinc finger E-box binding homeobox 1. ↑ increased; ↓ decreased.

**Table 2:** Nanoparticle-based delivery systems: examples of advantages and drawbacks.

Delivery System	Advantages	Drawbacks
Lipid-based nanoparticles	– Escape from mononuclear phagocyte system (MPS) uptake – Prolongation of circulating time – Enhanced permeability and retention time – Increased local drug levels	– Low encapsulation efficiency of small molecules – Cytotoxicity caused by cationic lipids – Systemic toxicity due to liver penetration
Polymer-based nanoparticles	– Facilitated incorporation of hydrophobic drugs – Increased stability compared to lipid-based ones	– Poor encapsulation for certain hydrophilic drugs – Insufficient toxicological assessments
Lipid–polymer hybrid nanoparticles	– High encapsulation efficiency – Well-defined release kinetics – Active targeted drug delivery – Well-tolerated serum stability	– Need to define the application in clinical practice – Need to identify hybrids with the highest quality and specific uses

## Data Availability

Not applicable.
